# Antimicrobial resistance genes and mobile genetic elements in compost amendments, soil, and the human gastrointestinal bacterial communities of gardeners during a single gardening season

**DOI:** 10.1128/msphere.00882-25

**Published:** 2026-06-04

**Authors:** Veronica R. Wirth, Shiyi Y. L. Xie, Madeleine M. Russell, Sihan Bu, Kameron Y. Sugino, Sarah F. Keller, Lixin Zhang, Katherine Alaimo, Alyssa W. Beavers, Sarah S. Comstock

**Affiliations:** 1 Department of Food Science and Human Nutrition, Michigan State University 437639, East Lansing, Michigan, USA; 2 Department of Epidemiology and Biostatistics, Michigan State University 172704, East Lansing, Michigan, USA; 3 Department of Microbiology & Molecular Genetics, Michigan State University 3078, East Lansing, Michigan, USA; 4 Department of Nutrition and Food Science, Wayne State University 675520, Detroit, Michigan, USA; University of Wisconsin-Madison, Madison, Wisconsin, USA

**Keywords:** soil, compost, human, resistome, microbiome, gardening, bacteria, antimicrobial resistance

## Abstract

The purpose of this study was to assess the antimicrobial resistance gene (ARG) and mobile genetic element (MGE) gene presence and abundance in soil, dairy manure and plant mixed compost (DMP), plant-based compost (P), and gardeners who used DMP across three time points. Soil (*n* = 1), DMP (*n* = 1), P (*n* = 1), and participant (*n* = 11) stool samples were collected at three time points during the 2019 gardening season in the Lansing, MI, area. DNA was extracted from each sample and stored at –20°C until use. Quantitative real-time PCR was used to detect ARGs and MGEs in each sample. Alpha diversity was assessed using richness, Shannon, and inverse Simpson indices. Shapiro-Wilk tests were used to assess normality. Beta diversity was assessed using Sorensen and Bray-Curtis dissimilarity indices. We detected a diverse composition of ARGs and MGEs in soil, compost, and human stool samples. DMP and soil exhibited greater ARG richness than P compost. In human stool, ARG and MGE profiles showed inter-participant variability but remained largely consistent within individuals across the gardening season. Alpha and beta diversity metrics revealed no significant shifts in the overall human gut resistome diversity across the gardening season. However, MGE gene composition differed significantly between DMP compost and human stool samples. Although ARG and MGE were detected in the soil and compost samples, there was no change in the ARG and MGE composition of gardeners’ gut resistomes over the gardening season. These findings suggest that short-term exposure to AMR-rich compost or soil may not substantially alter gut resistome structure in healthy adult populations.

**IMPORTANCE:**

Antimicrobial resistance is a growing public health concern, and compost and soil can contain genes that help bacteria resist antibiotics or share resistance with other bacteria. Many gardeners use manure-based compost, but it is unclear whether short-term exposure affects antimicrobial resistance in bacteria residing within the human gut. In this study, we detected antimicrobial resistance genes (ARGs) and mobile genetic elements (MGEs) in soil, two types of compost, and stool from gardeners across the 2019 gardening season near Lansing, MI. We found that manure-based compost and soil contained more ARGs than plant-based compost, but gardeners' gut resistomes remained stable over time. These results suggest that short-term compost exposure through gardening may not substantially alter the gut resistome.

## INTRODUCTION

Antimicrobial resistance (AMR) occurs when microorganisms, including bacteria, viruses, fungi, and parasites, no longer respond to drugs, making the treatment of infections more difficult and increasing the risk of disease transmission, serious illness, and disability (1). Humans have played a profound role in the development of AMR through overprescription of antimicrobial compounds in the medical and agricultural sectors (2). However, the problem of AMR is not limited to anthropogenic (human-related) activity. A growing body of research suggests that animals, soil, and bodies of water also play an important role in the origin, spread, and maintenance of AMR, making it a public health problem across ecosystems (3).

Antimicrobial resistance genes (ARGs) are specific genes present in microorganisms that make them resistant to the effect of antibiotics, and the term resistome refers to the set of all ARGs in a microorganism (4). Mobile genetic elements (MGEs), such as plasmids, transposons, and integrative elements, mediate gene transfer of ARGs to other microorganisms (5). AMR is one of the most pressing global health challenges under the “One Health” framework, an interdisciplinary, collaborative framework to achieve optimal health for people, animals, and the environment, due to the ability of AMR to spread rapidly in populations through multiple pathways, including the food chain, healthcare systems, and the natural environment (1, 6–8). The presence of AMR complicates the management of multiple infectious diseases in humans and animals (1).

Soil houses a diverse microbial community and is one of the largest known environmental reservoirs for bacteria harboring ARGs (9). Animal agriculture is a leading contributor to the accumulation of AMR within soils (10). Antibiotics are administered to livestock to prevent and treat diseases and promote growth (11), and the use of antibiotics in food-producing animals increased by 24% between 2009 and 2015 (12, 13). However, regulatory actions introduced by the Food and Drug Administration (FDA) have eliminated antimicrobial use for growth promotion and require veterinary approval before administration of antimicrobials. These regulations led to an initial decline in antimicrobial sales and use, followed by recent fluctuations and an uptick (14). Antibiotic-resistant bacteria can flourish in the gut microbial communities of livestock that repeatedly receive antibiotics through competition or horizontal gene transfer facilitated ARG acquisition (10, 12). Manure-based compost is a common soil amendment that is encouraged for soil health; however, application has been observed to increase ARG richness and abundance in soils (10). Raw livestock waste contains higher concentrations of ARGs, whereas composting processes can reduce their overall abundance (15). However, as reductions are often incomplete and some ARGs may persist, compost may still serve as a route for human exposure, although likely at lower levels than raw waste (15).

Human interaction with soil and manure-based compost is pronounced in gardening, where individuals frequently handle soil, consume home-grown produce, and spend extended periods outdoors (16). When gardeners apply manure-based composts to their soil and engage in regular garden tasks, they are exposed to microorganisms potentially harboring ARGs. Repeated exposures could increase the potential for ARG-carrying microbes to enter the gastrointestinal tract and interact with human gut microbiota (17). Through horizontal gene transfer, ARGs may be incorporated into the human gut metagenome, contributing to the spread of AMR (18).

Additional research is needed to examine gardeners’ exposure to AMR, including the potential impact of compost, a commonly used soil amendment, on AMR in the gastrointestinal tracts of gardeners. Our study investigated antimicrobial resistance, focusing on both ARGs and MGEs across different sample types (soil, compost, and human stool). We examined AMR in gardeners at three time points over a 5-month gardening season: before gardening (T1), after adding compost to their garden plots (T2), and at the peak of harvest (T3). Our study had two aims: (i) to examine ARGs and MGEs in compost containing dairy manure and plant-based materials (DMP), compost made solely of plant material (P), and soil, and (ii) to examine the ARGs and MGEs in gardeners who used DMP compost at the three time points and compare them with those in the DMP sample. We hypothesized that the resistomes of these three samples (soil, DMP, and P) would have distinct compositions and that the ARGs and MGEs in an individual’s gut microbiota would vary over time and differ from those in the DMP sample.

## MATERIALS AND METHODS

### Study design and sample collection

Participants were registered community gardeners who were gardening at one of the community gardens in the Greater Lansing Food Bank Garden Project network in metro Lansing, MI, or home gardeners registered to receive Garden Project support. Recruitment for this study occurred through attending garden orientation meetings and emailing enrolled gardeners. To be eligible for the study, participants must not have started gardening for the 2019 gardening season, must be non-pregnant, and must be between the ages of 18 and 70 years old. Upon enrollment, participants completed a survey that included questions on demographics.

**Fig 1:**
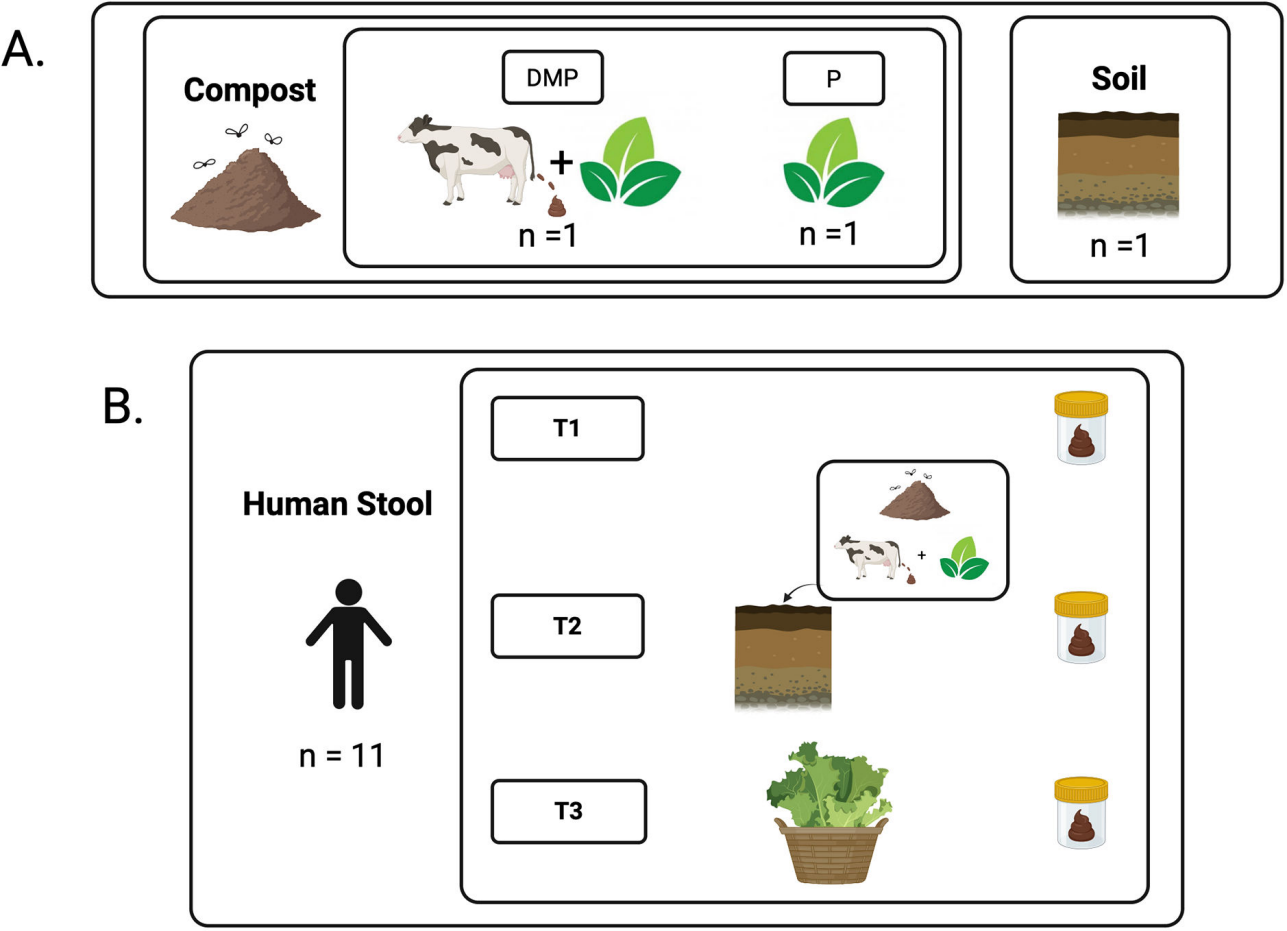
Study overview. (**A**) Three samples were analyzed for antimicrobial resistance genes (ARGs) and mobile genetic elements (MGEs): (i) compost generated from dairy manure and plant material (DMP; *n* = 1), (ii) compost from plant material (P; *n* = 1), and (iii) soil (*n* = 1). (**B**) Stool samples were collected by gardeners (*n* = 11) at three time points over a 5-month gardening season: before gardening (T1), after adding compost to their garden plots (T2), and at the peak of harvest (T3).

Twenty-nine participants were enrolled in the study. All enrolled participants were provided one of three compost types: dairy manure and plant-based compost (DMP compost, *n* = 11), chicken manure-based compost (*n* = 9), and plant-based compost (P compost, *n* = 9). Samples of two types of compost (DMP and P) were collected (Fig. 1A). P compost was included in this analysis to serve as a control to the DMP compost, as P compost, which is primarily plant material excluding animal-derived products, was hypothesized to contain fewer antimicrobial resistance genes than DMP. The dairy manure–plant mixed compost (DMP) was DairyDoo (Morgan Composting, Inc., Sears, MI), and the P was obtained from Hammond Farms (East Lansing, MI). Participants were instructed to apply compost as a ¼-inch surface layer, which corresponds to approximately one 40-lb bag per 50 square feet. Some results from this study have been published previously (16). Only the participants provided with the DMP compost were included in the human resistome analyses described herein. The DMP compost was prioritized, given its animal manure component and theoretically higher likelihood of containing antibiotic resistance genes.

Samples of two types of compost (DMP and P) were collected (Fig. 1A). DMP was a commercially available bagged compost made from composted dairy manure, dairy paunch manure, wood chips, and sawdust. Dairy paunch manure describes the contents within the rumen at the time of slaughter; it contains feed at various stages of digestion, including fermented and non-fermented feed and end products of microbial metabolism (19). The P compost was a locally produced, commercially available product made from food scraps and yard waste. Near the time of the T3 stool sample collection, soil samples were collected from each participant’s garden by a researcher. For each plot, six soil cores were taken to a depth of 4 inches and combined into a composite sample, from which DNA was extracted in four replicates. Equal amounts of gDNA from the four replicates were combined to generate a single gDNA sample per plot. Finally, equal amounts of gDNA from each plot were pooled to create one composite soil gDNA sample for AMR analysis. For each of the two types of compost, DMP and P, gDNA from four extractions of the source material was pooled.

Participants were provided with DMP compost and were instructed to spread the compost evenly over their garden plot and dig it into the top 6 inches of soil. During the gardening season, participants collected their stool samples at three time points (Fig. 1B). Time point 1 (T1) was prior to beginning gardening in May or June. At time point 2 (T2), participants were instructed to work the provided compost into their garden plots and spend at least 4 cumulative hours gardening prior to collecting this stool sample. The intent of the T2 time point was to collect samples after participants had begun active gardening, particularly plot preparation and planting. Because the amount of time required for these activities varies substantially across individuals, a minimum threshold of at least 4 cumulative hours of gardening was used to ensure a consistent, quantitative minimum level of exposure prior to T2. Time point 3 (T3), stool was collected during the peak of garden harvest in late August or early September. Each time participants submitted a stool sample, they completed a questionnaire that asked if they were currently taking antibiotics.

### DNA extraction and qRT-PCR

Stool, soil, and compost samples were stored at −80℃ until analysis. DNA was extracted using the DNeasy PowerSoil Pro Kit (Qiagen MoBio, Carlsbad, CA, USA) following the manufacturer’s instructions. The concentration of gDNA was quantified using the Quant-iT dsDNA Assay Kit (Invitrogen, Carlsbad, CA). After extracting DNA, AMR genes were quantified using the Takara SmartChip Real-time quantitative PCR (pPCR) platform. Primer sets targeting ARGs and MGEs were selected based on prior validated studies (20–26). The qPCR targets are listed in Table S1. Each reaction was performed in triplicate using SYBR Green 480 Master Mix (23). The thermal cycling protocol consisted of an initial step at 95°C for 2 min and 53 s, followed by 34 s at 95°C and 64 s at 60°C for each amplification cycle. To minimize false positives, a threshold cycle (Ct) cutoff of 30 was applied. Gene richness was defined as the number of ARGs or MGEs detected in each sample. Abundance was calculated by converting Ct values to gene copy numbers, which were then normalized to 16S rRNA gene levels.


$$\;\;\;\;\;\;\;\;\;\;\;\;\;\;GC=10^{\left(\frac{\left(30-Ct\right)}{3.33}\right)}$$

$$\;\;\;\;\;\;\;\;\;Normalized\;abundance=\frac{GC\left(ARG\right)}{GC\left(16SrRNA\right)}$$

### Data visualization and statistical analysis

Richness, inverse Simpson, and Shannon alpha diversity indices for various ARGs and MGEs in all sample types (stool, soil, and compost) were calculated in R (version 4.3.1) using the “vegan” package (27, 28). Shapiro-Wilk tests were used to test whether the alpha diversity data were normally distributed. For normally distributed data, repeated-measures one-way analysis of variance (ANOVA) with post hoc Tukey HSD was performed to examine differences in alpha diversity indices between the samples at a single time point. For non-normally distributed data, the Friedman test with post hoc Dunn test was used to examine differences in alpha diversity indices between the samples at a single time point. The indices for each type and sample were calculated using the “aggregate” function and visualized with a stacked bar plot using the “ggplot2” package (29). Separate bar plots for each type of ARG/MGE were generated using a loop to filter data by type, and all individual plots were combined into a single layout using the “patchwork” package for comprehensive comparison (30).

For all sample types (stool, soil, and compost), Beta diversity (Sorensen and Bray-Curtis dissimilarity matrix) was calculated with the “vegan” package in R and ordinated using principal coordinate analysis (PCoA). Permutational multivariate ANOVA (PERMANOVA) was performed to test the gene compositional differences between the participants at each time point based on the Sorensen or Bray-Curtis dissimilarity metrics using the adonis function in the “vegan” package (31). The percentages of shared genes across different time points and samples were calculated for each pair, and their distribution was visualized using box plots with the “ggplot2” and “ggbreak” packages. Heat maps visualizing the log abundance of each ARG class for both human and soil data sets were created using the functions “pivot_longer” and “ggplot” from R packages “tidyr” and “ggplot2” (32). Step plots and box plots were also generated using R, with data manipulation performed using the “dplyr” and “tidyr” packages (33), and with visualization using the “ggplot2” package. Custom plotting functions were used to display participant-level shifts in PCoA coordinates across time points. Euclidean distances between PCoA coordinates were calculated and visualized to assess temporal changes in ARG community structure and composition.

## RESULTS

### Participant characteristics

Overall demographics are presented in Table 1. Among the DMP participants, two were home gardeners, and the remainder were community gardeners from four different community gardens. Participants’ mean age was 34.7 ± 8.2 years (mean ± SD). Average BMI was 25 ± 5.8 (mean ± SD), and slightly over half of the participants were Asian (*n* = 6, 55%) and female (*n* = 6, 55%). Forty-five percent of participants had a household income between $30,000 and $49,999 (*n* = 5). No participants reported currently taking antibiotics at T1, participant 23 reported currently taking antibiotics at T2, and no participants reported currently taking antibiotics at T3. Participant 23 was not removed from our analysis at T2. Prior gardening experience varied among participants: six reported less than 1 year, 2 reported 1–2 years, and 3 reported 3–5 years of gardening prior to the 2019 season. Garden plot size was <200 ft² for five participants, 200–<500 ft² for one participant, and ≥500 ft² for three participants; plot size information was unavailable for two participants.

**TABLE 1:** Participant demographic characteristics^*a*^

Population characteristic	DMP
*N*	11
Continuous variables (mean ± SD)	
Age, yrs	34.7 ± 8.2
BMI, kg/m	25 ± 5.8
Categorical variables, *n* (%)	
Race	
Asian	6 (55)
Black	0
White	5 (45)
Sex	
Female	6 (55)
Male	5 (45)
Income	
Less than $10,000	1 (9)
Between $20,000 and $24,999	1 (9)
Between $25,000 and $29,999	1 (9)
Between $30,000 and $49,999	5 (45)
Between $50,000 and $74,999	3 (27)
Antibiotics, yes	
T1	0
T2	1 (10)
T3	0

^*a*^ SD, standard deviation; BMI, body mass index; T1, time point 1; T2, time point 2; and T3, time point 3.

### Normalized abundance of ARG and MGE

#### Soil and compost

**Fig 2:**
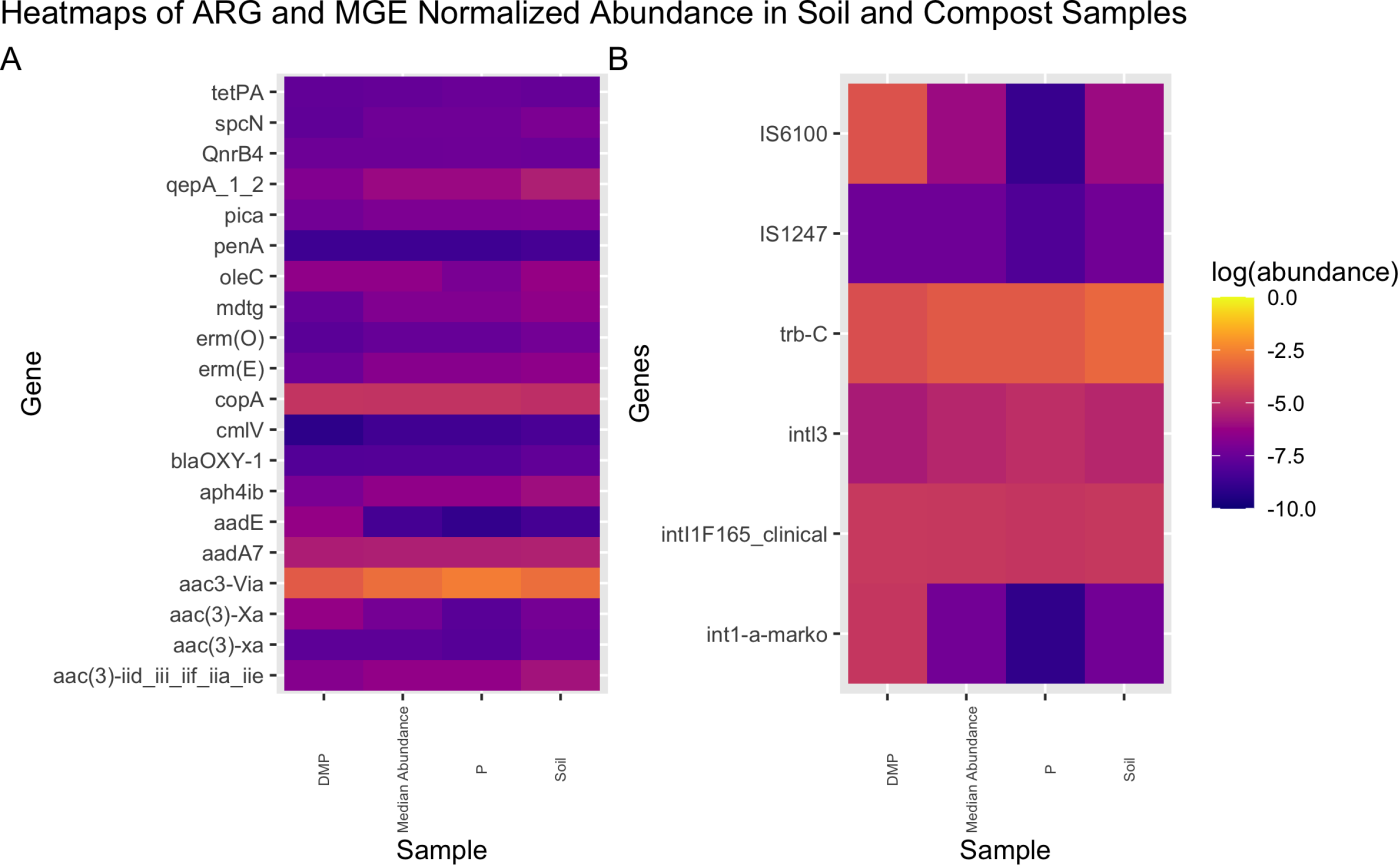
Heatmaps of the log-transformed normalized abundance of ARGs (**A**) and MGEs (**B**) in soil and compost samples. Samples include compost generated from DMP, compost from plant material only (P), and a composite garden soil sample. Median gene abundances across all samples are also shown (Median Abundance). Only genes with normalized abundance values greater than 0.0001 were included. Abundance was normalized to 16S rRNA gene copies and log₁₀-transformed. Warmer colors indicate higher relative abundance.

Heat maps of log-transformed normalized abundance values revealed the presence and normalized abundance of ARGs (Fig. 2A) and MGEs (Fig. 2B) in soil and compost samples. For ARGs, the highest log abundance was observed for genes *aac3-Via*, *copA*, and *aadA7*, which confer resistance to aminoglycosides. The abundances of these genes varied across samples (DMP, soil, and P compost). P compost and soil samples displayed high abundances for 17 out of 23 ARGs when compared to DMP. Some ARGs, including *qepA_1_2*, which confers resistance to fluoroquinolones; *mdtg*, conferring resistance to multiple drugs; *erm(E*), conferring resistance to MLSB; and *aph4ib*, *aadE*, and *aac(3)-Xa*, conferring resistance to aminoglycosides, displayed low but inconsistent abundances across the samples (Fig. 2A).

Regarding MGEs, the genes *trb-C*, *intl3*, and *intlF165_clinical* were consistently present across samples, with slight variation in abundance; *trb-C* abundance was the highest in soil and the lowest in DMP (Fig. 2B). These elements are associated with horizontal gene transfer: *trb-C* encodes a structural component of the type IV secretion system, *intl3* is an integron that allows bacteria to acquire new resistance traits, and *intlF165_clinical* is another integron that is strongly associated with multi-drug resistance. Other MGE-associated genes, such as *IS6100* and *int1-a-marko,* were most abundant in DMP compost (Fig. 2B). These values were notably higher in DMP than in soil or P compost, where abundance was lower.

#### Human stool

**Fig 3:**
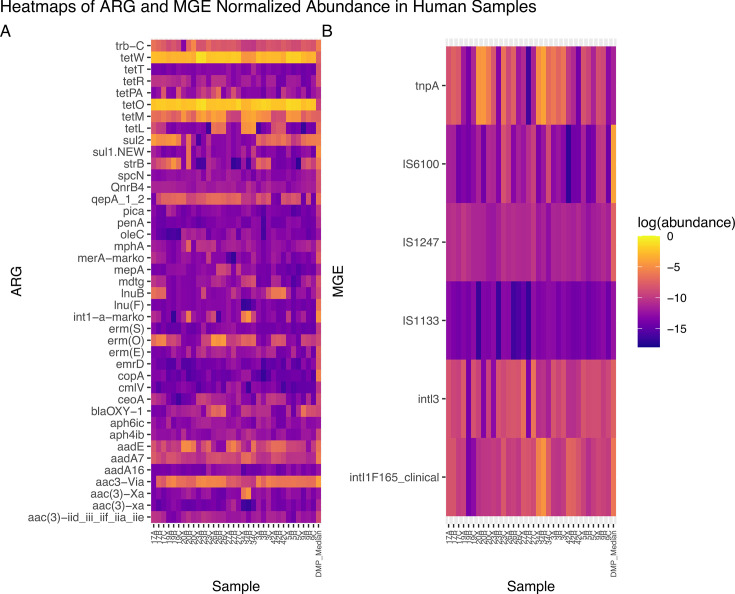
Heatmaps of the log-transformed normalized abundance of ARGs (**A**) and MGEs (**B**) in human stool samples from gardeners using a compost mixture of dairy manure and plant material (DMP). Sample IDs are shown on the *x*-axis, with time points labeled as A (baseline), B (peak season), and C (endpoint). Only genes with normalized abundance values greater than 0.0001 were included. Abundance values were normalized to 16S rRNA gene copies and log-transformed. Warmer colors indicate higher relative abundance. The DMP_Median column reflects the median log abundance of applicable genes across four replicate samples.

Heat maps of log-transformed normalized abundance values display the presence and normalized abundance of ARGs (Fig. 3A) and MGEs (Fig. 3B) in human stool samples from gardeners using the DMP amendment in their gardens alongside a reference column displaying the median abundance for each gene in DMP. Substantial inter-participant variability in ARG abundance was observed. Several genes exhibited participant-specific abundance patterns that were distinct from the rest of the cohort, including *tetM* and *tetL*, which confer resistance to tetracyclines; *qepA_1_2*, which confers resistance to fluoroquinolones; *lnuF*, which confers resistance to lincosamides; *aac(3)-Xa* and *aac(3)-xa*, which target gene variants for a gene that confers resistance to aminoglycosides. Notably, while these genes varied greatly between participants, their abundance levels were relatively consistent within individuals across time points. For example, participant 27 showed a consistently low abundance of *tetM* across all time points, whereas participant 34 exhibited consistently high levels of the same gene. However, the genes *tetO* and *tetW* were consistently highly detected between all participants across the gardening season. Interestingly, the *aac3-Via* gene, which confers resistance to aminoglycosides, was highly abundant in DMP and was also highly abundant in the human stool samples.

The heat map also revealed notable variation in MGE gene abundance across participants (Fig. 3B). Genes such as *intl1F165*, *IS6100*, and *IS1247* displayed distinct individual-level profiles, with relative consistency across time points. For instance, participant 34 exhibited consistently high abundance of *intl1F165*, while other participants showed markedly lower abundances. The MGE gene *tnpA* showed substantial variability both within and across participants, with the DMP compost sample exhibiting intermediate abundance. *IS6100* was consistently low in human samples but considerably higher in DMP. *IS1247* showed a similar, although less pronounced trend. *IS1133* remained low across most participants and was only slightly more abundant in the DMP compost.

### Alpha diversity

#### Human stool samples

The alpha diversity of ARG within the human stool samples was similar across time points (Table 2). Stool alpha diversity was compared by antibiotic class, with statistical testing performed across time points. Richness, Shannon, and inverse Simpson alpha diversity scores remained similar by class over time (Table 3). However, there were two exceptions. The richness of ARGs targeting fluoroquinolones decreased across time points, and the inverse Simpson index of tetracycline significantly decreased across time points.

**TABLE 2:** Alpha diversity of ARG in the human gut microbiota over a gardening season^*b*^

Index	T1	T2	T3	Overall	*P*-value^*a*^
Richness	31 (2.5)	31 (2.5)	31 (4.5)	31 (3)	0.966
Shannon	0.93 (0.22)	0.88 (0.19)	0.83 (0.19)	0.90 (0.24)	0.216
Inverse Simpson	2.09 (0.35)	2.07 (0.28)	1.93 (0.25)	2.07 (0.32)	0.148

^*a*^ Statistical comparisons were made using repeated measures one-way ANOVA test for parametric data and Friedman test for non-parametric data.

^*b*^ Data are presented as median (IQR). T1, time point 1; T2, time point 2; T3, time point 3.

**TABLE 3:** Alpha diversity of each ARG class of genes in the human gut microbiota over a gardening season^*b*^

Index	Class	Time point			*P*-value^*a*^
		T1	T2	T3	
Richness	Aminoglycoside	9 (0.5)	9 (1.5)	10 (1)	0.852
	Beta-lactams	2 (1)	1 (1)	2 (1)	0.513
	Fluoroquinolones	2 (1)	2 (0)	2 (0)	0.041
	MDR	4 (1)	4 (1)	4 (0.5)	0.495
	MLSB	5 (1)	6 (0.5)	6 (0)	0.519
	Sulfonamide	2 (1)	1 (1.5)	2 (1)	0.206
	Tetracycline	5 (1)	5 (1)	5 (0.5)	0.449
	MGE	7 (1.5)	6 (2)	6 (2)	0.315
	Other	2 (1)	2 (0)	2 (0)	0.223
Shannon	Aminoglycoside	0.40 (0.538)	0.41 (0.306)	0.57 (0.344)	0.148
	Beta-lactams	0.29 (0.397)	0.33 (0.556)	0.39 (0.457)	0.307
	Fluoroquinolones	0.09 (0.216)	0.18 (0.181)	0.10 (0.117)	0.307
	MDR	1.12 (0.433)	1.11 (0.541)	1.26 (0.805)	0.529
	MLSB	0.72 (0.895)	0.53 (0.862)	0.66 (0.612)	0.529
	Sulfonamide	0.39 (0.614)	0.70 (0.558)	0.62 (0.548)	0.078
	Tetracycline	0.75 (0.127)	0.69 (0.136)	0.67 (0.172)	0.178
	MGE	1.11 (0.369)	1.04 (0.433)	1.09 (0.291)	0.913
	Other	0.47 (0.473)	0.47 (0.479)	0.26 (0.405)	1.000
Inverse Simpson	Aminoglycoside	1.22 (0.774)	1.26 (0.237)	1.39 (0.602)	0.234
	Beta-lactams	1.18 (0.434)	1.22 (0.709)	1.29 (0.592)	0.307
	Fluoroquinolones	1.04 (0.142)	1.09 (0.124)	1.04 (0.066)	0.307
	MDR	2.35 (1.273)	2.43 (1.347)	3.01 (2.346)	0.913
	MLSB	1.97 (1.484)	1.45 (1.175)	1.37 (0.561)	0.336
	Sulfonamide	1.30 (0.89)	2.01 (0.97)	1.59 (0.93)	0.078
	Tetracycline	2.00 (0.094)	1.86 (0.272)	1.79 (0.36)	0.021
	MGE	2.52 (1.013)	2.43 (1.313)	2.44 (0.652)	0.761
	Other	1.41 (0.738)	1.42 (0.631)	1.15 (0.555)	1.000

^*a*^ Statistical comparisons were made using repeated-measures one-way ANOVA test for parametric data, and Friedman test with Dunn test for non-parametric data. *P* values were not FDR-corrected.

^*b*^ Time point data are presented as median (IQR). T1, time point 1; T2, time point 2; T3, time point 3; MDR, multi-drug resistance; MLSB, macrolide-lincosamide-streptogramin B resistance; MGEs, mobile genetic elements.

#### Compost and soil samples

Alpha diversity of ARGs in soil (T3) and compost samples was assessed using single, composite samples from quadruple DNA extractions of each plot or sample type. The following alpha diversity metrics were calculated: richness, Shannon, and inverse Simpson indices (Table 4). Soil samples exhibited the highest ARG richness (141), while P compost had the lowest (97). Shannon diversity, which is responsive to rare genes, was also the highest in soil (0.69) and the lowest in P compost (0.58). In contrast, the inverse Simpson index, a measure of richness in a dataset with uniform evenness, was the highest in P compost (2.11) and the lowest in DMP compost (1.71).

**TABLE 4:** Alpha diversity of each of the two composts and the soil sample^*b*^

	DMP	P	Soil
Richness ARG^*a*^	123	97	141
Shannon ARG^*a*^	0.66	0.58	0.69
Inverse Simpson ARG^*a*^	1.71	2.11	1.78

^*a*^ No statistical comparisons were made due to the use of a single composite sample for each sample type.

^*b*^ ARG, antimicrobial resistance gene; DMP, compost containing dairy manure and plant-based materials; P, compost made solely of plant material.

Across antimicrobial classes (Table 5), soil generally exhibited the highest richness, particularly for aminoglycosides, beta-lactams, and vancomycin. Fluoroquinolone ARGs were consistently low in richness across sample types, but their diversity was the highest in soil, based on the Shannon index, and in DMP compost, based on the inverse Simpson index. P compost consistently showed the lowest richness for multiple classes, including aminoglycoside, MLSB, and tetracycline. Notably, vancomycin ARGs displayed strikingly high diversity in DMP (inverse Simpson = 21.01) compared to P compost and soil.

**TABLE 5:** Alpha diversity indices of antimicrobial resistance gene (ARG) classes in compost and soil samples^*b*^

	Richness index^*a*^			Shannon index^*a*^			Inverse Simpson index^*a*^		
	DMP	P	Soil	DMP	P	Soil	DMP	P	Soil
Aminoglycoside	37	29	40	0.0445	0.0817	0.0700	2.31	1.31	2.02
Beta-lactam	12	13	19	0.0015	0.0017	0.0027	12.92	9.05	12.76
Fluoroquinolone	3	4	3	0.0019	0.0029	0.0046	2.21	1.73	1.39
MDR	17	17	18	0.0100	0.0119	0.0102	1.49	2.21	2.23
MGE	19	19	30	0.0791	0.0455	0.0656	5.04	2.35	2.38
MLSB	23	13	21	0.0071	0.0051	0.0079	8.23	6.91	6.34
Other	11	10	15	0.5747	0.4744	0.5909	1.30	1.46	1.31
Sulfonamide	3	2	2	0.0163	0.0001	0.0014	2.25	3.83	1.86
Tetracycline	11	3	11	0.0040	0.0011	0.0023	6.32	3.57	7.74
Vancomycin	6	6	12	0.0004	0.0010	0.0010	21.01	6.43	8.25

^*a*^ No statistical comparisons were made due to the use of a single composite sample for each sample type.

^*b*^ DMP, compost containing dairy manure and plant-based materials; P, compost made solely of plant material; MDR, multi-drug resistance; MLSB, macrolide-lincosamide-streptogramin B resistance; MGEs, mobile genetic elements.

MGEs exhibited the highest richness in soil samples compared to DMP and P compost (Table 5). However, MGE diversity, as measured by the Shannon and inverse Simpson indices, was the highest in DMP.

### Beta diversity: human gastrointestinal tract resistome

#### Compost versus human stool resistomes

**Fig 4:**
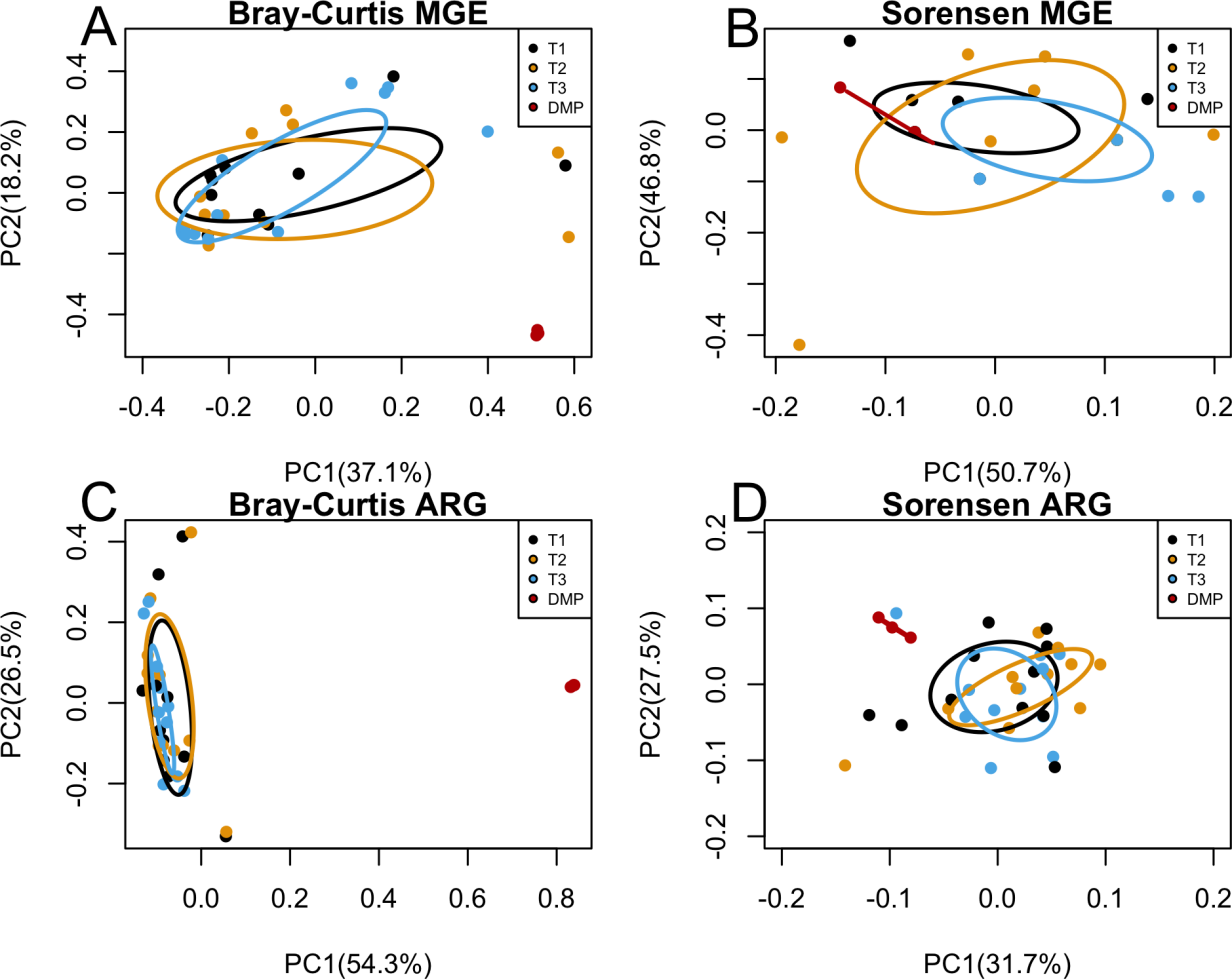
Beta diversity of gardener’s gut resistomes and the DMP compost resistome. Principal coordinates analysis (PCoA) plots based on Bray-Curtis and Sorensen dissimilarities show the compositional variation in mobile genetic elements (MGEs) and antimicrobial resistance genes (ARGs) across three time points (T1–T3) and in DMP compost. (**A**) Bray-Curtis dissimilarity of MGE (PERMANOVA   <  0.001; PERMDISP  =  0.015). (**B**) Sorensen dissimilarity of MGE (PERMANOVA   =  0.17; PERMDISP  =  0.11). (**C**) Bray-Curtis dissimilarity of ARG (PERMANOVA   <  0.001; PERMDISP  =  0.31). (**D**) Sorensen dissimilarity of ARG (PERMANOVA  =  0.009; PERMDISP  =  0.012). Ellipses represent one standard deviation (SD) of dispersion within each group, centered around the group centroid in the ordination space. Abbreviations: DMP, compost containing dairy manure and plant-based materials; T1, time point 1; T2, time point 2; T3, time point 3.

There was a significant difference in the structure of the bacterial MGE between all human stool microbiota time points compared to DMP (*P* < 0.001, Fig. 4A). There was a non-significant difference in the bacterial MGE composition between time points and DMP (*P* = 0.17, Fig. 4B). There was a significant difference in the structure of the bacterial ARG between all human stool microbiota time points compared to DMP (*P* < 0.001, Fig. 4C). There was a significant difference in the bacterial ARG composition between all human stool microbiota time points compared to DMP (*P* = 0.009, Fig. 4D).

#### Human stool resistomes over the course of a single gardening season

**Fig 5:**
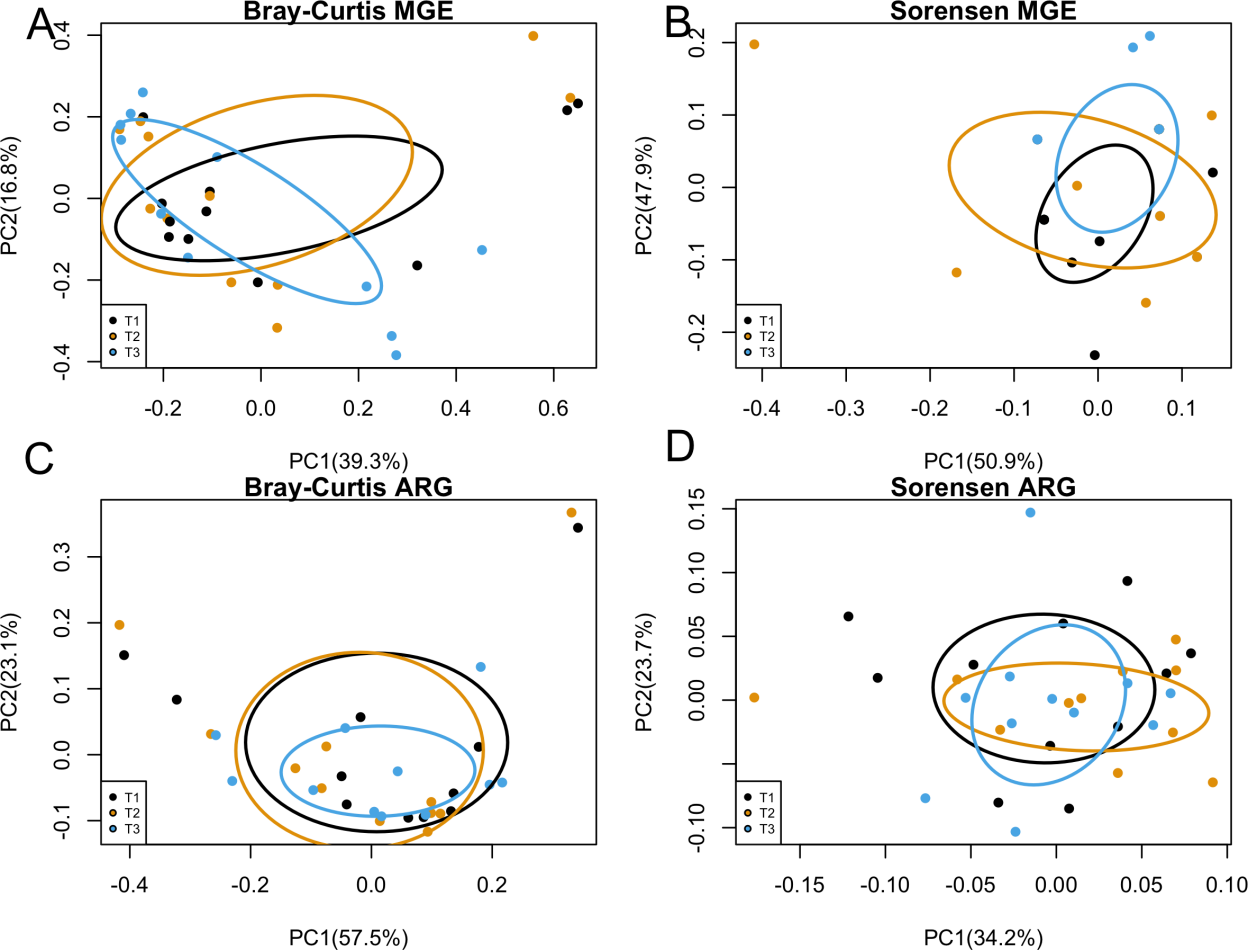
Beta diversity of gardener gut resistomes over the course of a single gardening season without DMP compost resistomes plotted. (**A**) Bray-Curtis dissimilarity of MGEs (PERMANOVA  =  1.0; PERMDISP  =  0.23). (**B**) Sorensen dissimilarity of MGEs (PERMANOVA  =  0.24; PERMDISP  =  0.23). (**C**) Bray-Curtis dissimilarity of ARGs (PERMANOVA  =  0.44; PERMDISP  =  0.74). (**D**) Sorensen dissimilarity of ARGs (PERMANOVA  =  0.19; PERMDISP  =  0.68). Ellipses represent one standard deviation (SD) of dispersion within each group, centered around the group centroid in the ordination space. Abbreviations: ARG, antimicrobial resistance gene; MGE, mobile genetic element; T1, time point 1; T2, time point 2; T3, time point 3; DMP, compost containing dairy manure and plant-based materials.

The structure (Fig. 5A and C) and composition (Fig. 5B and D) of the MGE (Fig. 5A and B) and ARG (Fig. 5C and D) beta-diversity in stool samples collected from gardeners who had amended their soil with DMP were similar between time points (*P* > 0.05).

### Beta diversity step plots: human stool over time

**Fig 6:**
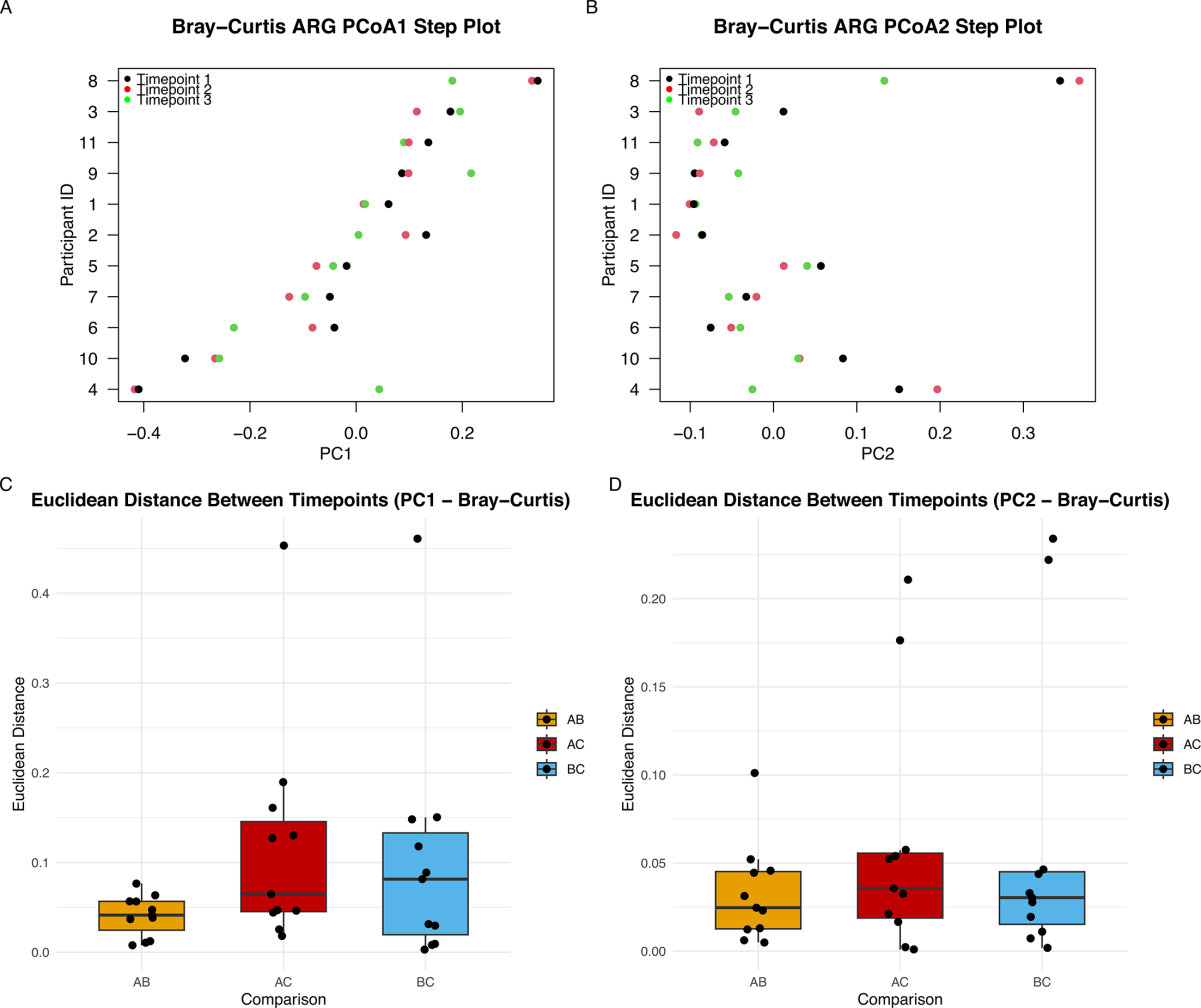
Beta diversity (Bray-Curtis) of ARG profiles in stool samples from gardeners using DMP compost across three time points: A (T1), B (T2), and C (T3). (**A**) Step plot of beta diversity (Bray-Curtis) distances along PCoA1. (**B**) Step plot of beta diversity (Bray-Curtis) distances along PCoA2. (**C**) Euclidean distances between time points for each participant along PCoA1 (*P* = 0.34). (**D**) Euclidean distances between time points for each participant along PCoA2 (*P* = 0.76). Distances were compared by time point groups using the Friedman test. In panels C and D, the center line represents the median, the box spans the IQR, whiskers extend to 1.5 times the IQR, with individual points representing each participant’s Bray-Curtis distance along the designated PCoA between time points. ARG, antimicrobial resistance gene.

**Fig 7:**
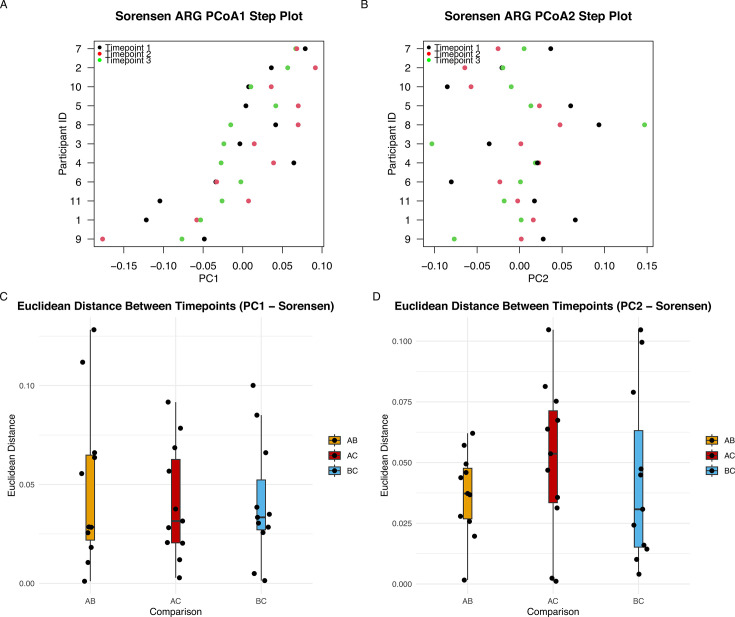
Beta diversity (Sorensen) of ARG profiles in stool samples from gardeners using DMP compost across three time points: A (T1), B (T2), and C (T3). (**A**) Step plot of beta diversity (Sorensen) distances along PCoA1. (**B**) Step plot of beta diversity (Sorensen) distances along PCoA2. (**C**) Euclidean distances between time points for each participant along PCoA1 (*P* = 0.76). (**D**) Euclidean distances between time points for each participant along PCoA2 (*P* = 0.15). Distances were compared using the Friedman test. In panels C and D, the center line represents the median, the box spans the IQR, whiskers extend to 1.5 times the IQR, with individual points representing each participant’s distance between time points. ARG, antimicrobial resistance gene.

The overall structure (Fig. 6) and composition (Fig. 7) of the ARG repertoire remained similar across time points, with no consistent directional shifts observed along either PC1 (Fig. 6A and 7A) or PC2 (Fig. 6B and 7B). While participant-specific variation was evident, the extent of change between time points was comparable across PCoA1 (Fig. 6C and 7C) and PCoA2 (Fig. 6D and 7D) axes for both dissimilarity indices (Bray-Curtis, Sorensen), with no statistically significant differences detected. Notably, for Bray-Curtis beta diversity, the ARG repertoire of the same participant demonstrated shifts larger than 0.4 units from T1 to T3 and from T2 to T3 on PCoA1 (Fig. 6C). A single participant demonstrated shifts larger than 0.2 units on PCoA2 (Fig. 6D) from both T1 to T3 and T2 to T3. While another participant demonstrated shifts larger than 0.2 units from T2 to T3 alone. For Sorensen beta diversity, the ARG repertoire of two participants demonstrated shifts larger than 0.09 units from T1 to T2, but only one demonstrated a shift larger than 0.09 units from T2 to T3 on PCoA1 (Fig. 7C). A single participant demonstrated a shift larger than 0.09 units on PCoA2 (Fig. 7D) from T1 to T3. Two participants demonstrated shifts larger than 0.09 units on PCoA2 (Fig. 7D) from T2 to T3.

### Shared genes in the human gastrointestinal tract resistome across the gardening season

**Fig 8:**
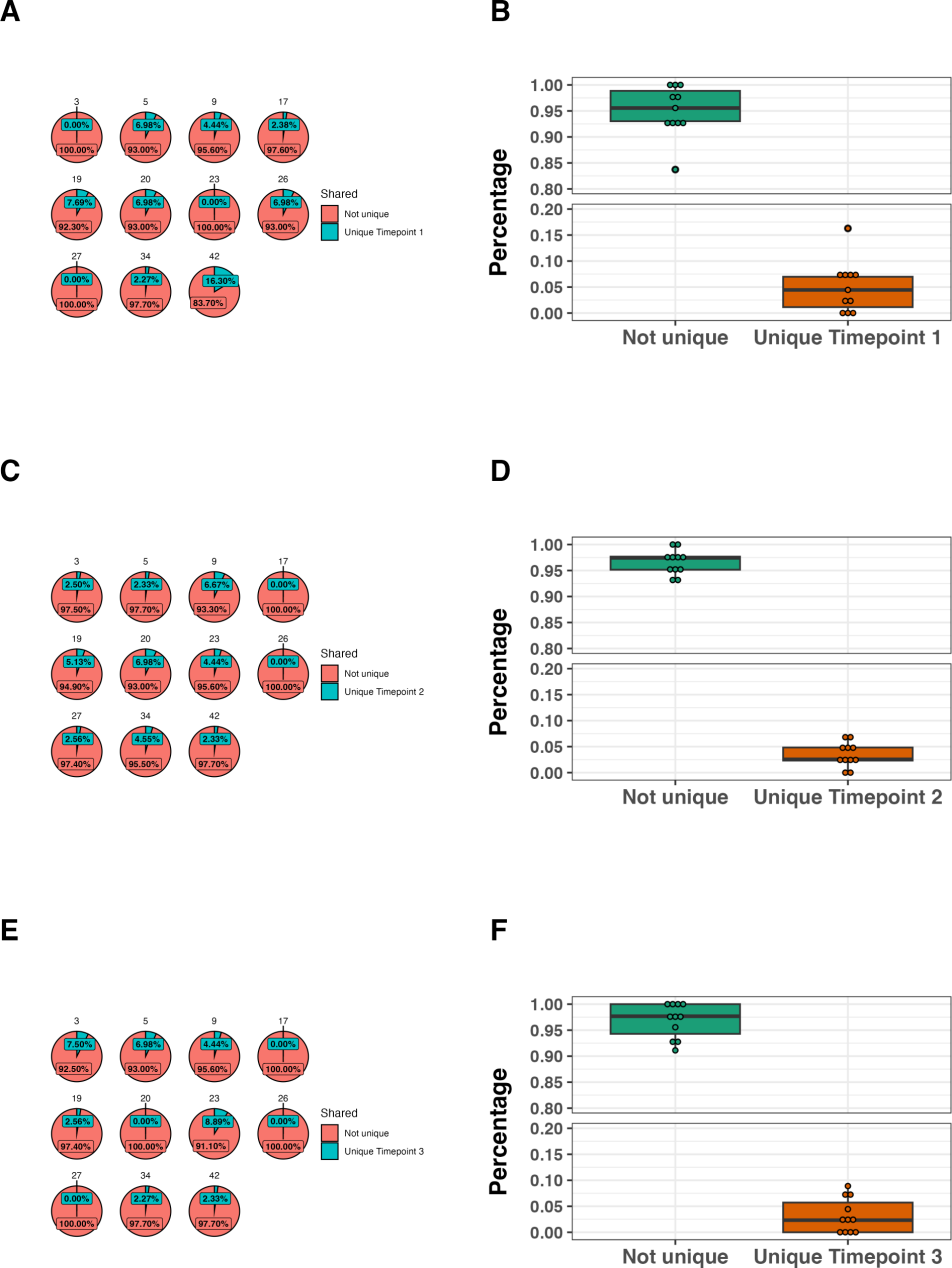
Shared ARG/MGE by time point (T1, T2, and T3). Pie charts showing the proportion of shared (red) and unique (blue-green) genes at T1 (**A**), T2 (**C**), and T3 (**E**), respectively. Box plots showing the percentage of shared and unique genes at T1 (**B**), T2 (**D**), and T3 (**F**). The line in the box is the median value, and the box depicts the 25th to 75th interquartile range having whiskers from min to max with outliers as dots outside the whiskers. Shared genes (not unique) consistently accounted for a high proportion (median ≈ 95%–97%), while unique genes were less prevalent (median ≈ 3%–5%). The overall difference in gene proportions across time points was not statistically significant (*P* = 0.33). Abbreviations: ARG, antimicrobial resistance gene; MGE, mobile genetic element.

For each participant, the percentage of shared genes, that is, not unique, was consistently high across all time points (Fig. 8), with median values close to 95% and unique genes prevalence occurring at a relatively low level, with median values around 3%–5%. For shared genes, at T1, an average of 95% genes were shared. At T2, an average of 96.6% genes were shared, and at T3, an average of 96.8% genes were shared. The percent of genes unique to T1 (average = 4.9%) was numerically greater than that for T2 (average = 3.4%) or T3 (average = 3.2%). The overall shared and unique gene proportions were similar across the three time points (*P* = 0.33). The gene classes (Table 6) and specific genes (Table 7) unique to at least one participant’s gastrointestinal resistome at each time point are presented.

**TABLE 6:** Gene classes unique to each time point in at least one participant’s gastrointestinal resistome^*a*^

Unique T1	Unique T2	Unique T3
MDR	–	MDR
–	Amphenicol	–
–	Beta-Lactam	–
–	Fluoroquinolone	Fluoroquinolone
–	Amphenicol	Amphenicol
Sulfonamide	Sulfonamide	Sulfonamide
Tetracycline	Tetracycline	Tetracycline
MLSB	MLSB	MLSB
MGEs	MGEs	MGEs
Aminoglycoside	Aminoglycoside	Aminoglycoside

^*a*^ MDR, multi-drug resistance; MLSB, macrolide-lincosamide-streptogramin B resistance; MGEs, mobile genetic elements; T1, time point 1; T2, time point 2; and T3, time point 3. "–" indicates that the corresponding gene class was not detected in at least one participant’s gastrointestinal resistome at the referenced timepoint.

**TABLE 7:** Genes unique to each time point in at least one participant’s gastrointestinal resistome^*a*^

Unique T1	Unique T2	Unique T3
–	strB	strB
mepA	–	mepA
merA-marko	–	merA-marko
–	aac(3)-Xa	–
sul1 NEW	sul1 NEW	–
–	int1-a-marko	int1-a-marko
lnuB	lnuB	lnuB
–	tetT	tetT
–	–	aph4ib
–	–	aac(3)-iid_iii_iif_iia_iie
aadE	–	–
aph6ic	–	–
–	qepA_1_2	qepA_1_2
–	ceoA	ceoA
IS6100	IS6100	–
erm(E)	erm(E)	–
IS1133	IS1133	–
tetL	–	tetL
tetR	–	tetR
–	–	erm(O)
int1-a-marko	–	–
–	cmlV	–
–	penA	–
intI1F165_clinical	–	–
–	–	mphA

^*a*^ T1, Time point 1; T2, Time point 2; T3, Time point 3. "–" indicates the corresponding gene class was not detected in at least one participant’s gastrointestinal resistome at the referenced timepoint.

## DISCUSSION

This study investigated the resistomes of soil, DMP, P, and gardeners’ gut microbiota, as well as the association between exposure to DMP and AMR in gardeners’ gut microbiota. Overall, soil and P compost exhibited higher ARG abundance than DMP for the majority of genes, although the abundance of some ARGs varied greatly. In soil and compost samples, the most abundant ARGs were *aac(3)-Via*, *copA*, and *aadA7*, which confer resistance to aminoglycosides. Several MGEs, including *trb-C*, *intlF165_clinical*, and *intl3*, were detected consistently across soil and compost samples. Soil samples had the highest ARG richness and Shannon diversity scores, while P compost had the lowest. MGE richness was also highest in the soil. When analyzing the human samples, ARG and MGE abundance varied between participants but remained relatively consistent within individuals over time. High inter-participant variability was observed for several genes, including *tetM*, *qepA_1_2*, and *aac3-Via*. Alpha diversity metrics of human stool samples remained stable across time points. The percentage of shared genes was consistently high (~95%). Notably, ARG and MGE profiles differed between compost and human stool samples.

ARGs and MGEs have been widely reported in soil and manure-amended compost, with agricultural soils and organic amendments acting as reservoirs for ARGs and MGEs (34–36). Manure-based amendments can introduce exogenous ARGs and MGEs, thereby increasing AMR in soil microbes (10). Our study noted the presence of numerous ARGs and MGEs within soil samples collected primarily from urban community gardens. Similarly, one study evaluating the soil microbiome of Detroit urban community gardens observed the presence of ARGs such as *tetM*, *sul1*, and *aac3-Via*, and MGEs including *intl1* and *IS6100*—all of which were present in our composite soil sample (37). Future investigations comparing soil samples collected from urban and rural sites could elucidate differences in ARG and MGE compositions and abundance between urban and rural soils, illustrating AMR burden in both environments.

Importantly, despite the routine exposure to an AMR reservoir through gardening, we observed that the human gut resistome was stable across the gardening season, with no significant shifts in ARG or MGE diversity or structure. This finding contrasts with Mahmud et al. (38), who observed higher ARG loads in those who regularly work and interact directly with livestock compared to unexposed controls, emphasizing that prolonged and intensive exposure may be key factors in gut resistome alteration (38). Additionally, Guo et al. (20) reported notable overlaps in ARG profiles between poultry and the humans that raise them in rural Ecuador, where frequent and close contact with poultry and their waste products was found to facilitate the acquisition of ARGs into the human gut resistome (20). In contrast, the lack of differences in ARG and MGE diversity and structure across the gardening season within this cohort of gardeners suggests that short-term, low-intensity exposure to dairy manure through gardening may not be sufficient to alter the gut resistome. Notably, the gardeners were not working directly with livestock or their waste. While certain ARGs like *aac3-Via* were found in both compost and stool samples, there was limited evidence for transmission or acquisition of these genes over the study period, as genes shared within gardeners’ guts were similar across all three time points. The home and community gardening settings, with less frequent or intense exposure to manure-based amendments, may explain the absence of such overlap.

By assessing stool samples longitudinally over the gardening season, this study demonstrated that the gardeners’ gut resistome remained stable, despite the presence of diverse ARGs and MGEs in soil and compost. Sampling at three time points (before gardening, after compost application, and during peak harvest) enabled longitudinal assessment of ARG and MGE profiles. In addition, quantitative real-time PCR enabled sensitive, gene-specific detection, with relative abundance calculated from Ct-derived gene copy numbers and normalized to 16S rRNA gene levels to account for differences in total bacterial abundance across samples.

There are some limitations to this study. The relatively small sample size of 11 seasonal gardeners from a single area limits the generalizability of the findings. Environmental sampling included soil collected from multiple gardens and two types of compost, and no measurements were made of other physicochemical characteristics (e.g., pH or heavy metals) that may affect ARG persistence and distribution (17, 39). In addition, the qRT-PCR targeted a specific set of ARGs and MGEs and may have missed other relevant or novel resistance genes. The lack of a non-gardening control group prohibits the examination of seasonal changes in the gut resistome that may occur independent of gardening exposure. In addition, participants reported varying levels of prior gardening experience, and pre-existing resistome differences related to past gardening practices cannot be excluded. However, in a sensitivity analysis comparing participants with less than 1 year versus more than 1 year of prior gardening experience, no significant differences in baseline gut resistome profiles were observed (data not shown). Baseline soil sampling prior to compost application and prior to peak harvest was not performed, which limits characterization of seasonal changes in soil resistomes over the gardening season. Information on garden characteristics, including plant types and biocide use, was not collected and therefore could not be analyzed. These factors may influence the gut microbiome and resistome and could confound observed associations. In addition, although dietary habits may influence the composition of the gut microbiota, dietary intake was not accounted for in this analysis. Finally, participants were not asked about recent livestock exposure, which may have excluded an unmeasured source of ARG exposure. As livestock exposure has been associated with human gut resistome acquisition of ARGs, the absence of this information limits our ability to fully account for non-gardening related ARG exposures (20).

This study contributes to the growing body of research on the environmental reservoir of antimicrobial resistance with potential relevance to human health and the importance of soil health for human health (3, 40, 41). Although high concentrations of ARGs and MGEs were detected in soil and compost, the results of the study suggest that short-term exposure through seasonal gardening activities may have a limited impact on the composition of the human gut resistome. The data presented here suggest that for individuals engaged in community or home gardening under similar conditions, the risk of acquiring additional ARGs from soil or compost exposure during community or home gardening is low.

Future studies should include larger and more diverse populations across geographic regions and seasons. Integration of functional assessments (e.g., metagenomic sequencing or phenotypic resistance testing) is critical to understand whether detected genes are active and/or transferable. In addition, future studies should also incorporate behavioral data, such as diet, time in contact with soil, and hygiene practices, to better understand the context of exposure risk. Inclusion of control groups and extended follow-up periods will further clarify the dynamic relationship between environmental AMR sources and human gut antimicrobial resistance.

### Conclusion

This study characterized ARG and MGE profiles in soil, compost, and stool samples from seasonal gardeners over a single growing season. Despite high ARG and MGE richness in environmental samples, human gut resistome composition remained largely stable, with limited within-person changes observed. These findings suggest that short-term exposure to AMR-rich compost or soil may not substantially alter gut resistome structure in healthy adult populations.

## SUPPLEMENTAL MATERIAL

Table S1 msphere.00882-25-s0001.docx Genes included in the analyses.

ASM does not own the copyrights to Supplemental Material that may be linked to, or accessed through, an article. The authors have granted ASM a non-exclusive, world-wide license to publish the Supplemental Material files. Please contact the corresponding author directly for reuse.
