# Supplementary material for: Antimicrobial resistance genes and mobile genetic elements in compost amendments, soil, and the human gastrointestinal bacterial communities of gardeners during a single gardening season
Source: mSphere. 2026 Jun 4;11(6):e00882-25. doi: 10.1128/msphere.00882-25 (PMC13317230; doi:10.1128/msphere.00882-25)
Supplement: Table S1 — Genes included in the analyses. [file msphere.00882-25-s0001.docx]

Supplemental Table 1. List of genes included in the analyses.

| Assay No. | Gene | Class | Assay No. | Gene | Class |
| --- | --- | --- | --- | --- | --- |
| 1 | 16S 1 | 16s_RNA_gene | 25 | Tp614 | MGEs |
| 2 | 16S 2 | 16s_RNA_gene | 26 | IS613 | MGEs |
| 3 | aacC2 | Aminoglycoside | 201 | tnpA | MGEs |
| 6 | aacA/aphD | Aminoglycoside | 202 | tnpA | MGEs |
| 8 | aac(6')-II | Aminoglycoside | 203 | tnpA | MGEs |
| 14 | aphA3 | Aminoglycoside | 204 | tnpA | MGEs |
| 49 | sat4 | Aminoglycoside | 205 | tnpA | MGEs |
| 95 | aac(6')-Ib | Aminoglycoside | 206 | tnpA | MGEs |
| 97 | aadA2 | Aminoglycoside | 207 | tnpA | MGEs |
| 98 | aadA5 | Aminoglycoside | 336 | int1-a-marko | MGEs |
| 104 | aph(2')-Id | Aminoglycoside | 338 | intl2 | MGEs |
| 151 | aadD | Aminoglycoside | 340 | IncN_rep | MGEs |
| 168 | aadA9 | Aminoglycoside | 342 | IncP_oriT | MGEs |
| 170 | aphA1 | Aminoglycoside | 343 | IncQ_oriT | MGEs |
| 174 | aadE | Aminoglycoside | 358 | trfa | MGEs |
| 175 | str | Aminoglycoside | 359 | intI1F165_clinical | MGEs |
| 176 | strA | Aminoglycoside | 366 | orf39-IS26 | MGEs |
| 400 | aac3ia | Aminoglycoside | 369 | ISPps1-pseud | MGEs |
| 402 | aph4ib | Aminoglycoside | 370 | ISSm2-Xanthob | MGEs |
| 403 | aph3via | Aminoglycoside | 371 | ISAba3-Acineto | MGEs |
| 404 | aph6ic | Aminoglycoside | 372 | ISEfm1-Entero | MGEs |
| 405 | ArmA | Aminoglycoside | 376 | IS1111 | MGEs |
| 406 | spcN | Aminoglycoside | 380 | pAKD1-IncP-1β | MGEs |
| 407 | spec_aph | Aminoglycoside | 381 | pBS228-IncP-1α | MGEs |
| 408 | aac(3)-ib | Aminoglycoside | 383 | IS1133 | MGEs |
| 409 | aac(3)-id_ie | Aminoglycoside | 1522 | intl3 | MGEs |
| 410 | aac(3)-iid_iii_iif_iia_iie | Aminoglycoside | 1524 | mobA | MGEs |
| 411 | aac(3)-xa | Aminoglycoside | 1525 | traN | MGEs |
| 412 | Aac6-Aph2 | Aminoglycoside | 1526 | tra-A | MGEs |
| 413 | aac(6)-ig | Aminoglycoside | 1527 | trb-C | MGEs |
| 414 | aac(6)-iic | Aminoglycoside | 1528 | ISCR1 | MGEs |
| 415 | aac(6)-ij | Aminoglycoside | 1546 | IS26 | MGEs |
| 417 | aac(6)-im | Aminoglycoside | 1547 | IS3 | MGEs |
| 418 | aac(6)-ir | Aminoglycoside | 1548 | IS256 | MGEs |
| 419 | aac(6)-is_iu_ix | Aminoglycoside | 1550 | ISEcp1 | MGEs |
| 420 | aac(6)-iv_ih | Aminoglycoside | 1551 | IS200 | MGEs |
| 421 | aac(6)-iw | Aminoglycoside | 1552 | IS1247 | MGEs |
| 422 | aac(6)-iz | Aminoglycoside | 1553 | IS630 | MGEs |
| 423 | aacA43 | Aminoglycoside | 1556 | TN5403 | MGEs |
| 424 | aadA6 | Aminoglycoside | 1557 | IS200 | MGEs |
| 425 | aadA7 | Aminoglycoside | 1558 | IS21-ISAs29 | MGEs |
| 426 | aadA10 | Aminoglycoside | 1559 | Tn3 | MGEs |
| 427 | aadA16 | Aminoglycoside | 1560 | IS6/257 | MGEs |
| 428 | aadA17 | Aminoglycoside | 1561 | IS6100 | MGEs |
| 429 | aadB | Aminoglycoside | 1562 | IS15DI | MGEs |
| 430 | ant4-ib | Aminoglycoside | 1563 | IncN_korA | MGEs |
| 431 | ant6-ia | Aminoglycoside | 1564 | lncF_FIC | MGEs |
| 432 | aph3-ib | Aminoglycoside | 1565 | IncI1_repI1 | MGEs |
| 433 | aph3-viia | Aminoglycoside | 1566 | IncHI2-smr0018 | MGEs |
| 434 | aph4-ia | Aminoglycoside | 1567 | IS91 | MGEs |
| 435 | aph(3'')-ia | Aminoglycoside | 1568 | IS5/IS1182 | MGEs |
| 436 | apmA | Aminoglycoside | 1569 | cro | MGEs |
| 437 | aph_viii | Aminoglycoside | 1570 | EAE_05855 | MGEs |
| 438 | acc3-iva | Aminoglycoside | 17 | ermK | MLSB |
| 1501 | aac(6')I1 | Aminoglycoside | 73 | vatD | MLSB |
| 1502 | aac(6')-Iy | Aminoglycoside | 91 | erm(36) | MLSB |
| 1503 | aph6ia | Aminoglycoside | 137 | ermT | MLSB |
| 1510 | aac3-Via | Aminoglycoside | 138 | msr(C) | MLSB |
| 1540 | aph3-III | Aminoglycoside | 141 | mphB | MLSB |
| 1541 | ant6-ib | Aminoglycoside | 149 | msr(A) | MLSB |
| 1545 | aac(3)-Xa | Aminoglycoside | 209 | ermX | MLSB |
| 51 | catB3 | Amphenicol | 225 | vatC | MLSB |
| 52 | catB8 | Amphenicol | 227 | vgaB | MLSB |
| 53 | ceoA | Amphenicol | 229 | pica | MLSB |
| 127 | cmlA1 | Amphenicol | 251 | lnuA | MLSB |
| 129 | cmx(A) | Amphenicol | 252 | lmrA | MLSB |
| 130 | catA1 | Amphenicol | 263 | vat(E) | MLSB |
| 375 | cmlA5 | Amphenicol | 268 | vgbB | MLSB |
| 900 | cat | Amphenicol | 270 | ermY | MLSB |
| 902 | catA2 | Amphenicol | 275 | erm(C) | MLSB |
| 903 | catA3 | Amphenicol | 277 | cfr | MLSB |
| 904 | catB2 | Amphenicol | 283 | ermA/ermTR | MLSB |
| 906 | catB9 | Amphenicol | 285 | oleC | MLSB |
| 908 | cat(pC221) | Amphenicol | 288 | carB | MLSB |
| 909 | catP | Amphenicol | 289 | ermZ/pikR1 | MLSB |
| 910 | catQ | Amphenicol | 801 | ere(A) | MLSB |
| 911 | cmlV | Amphenicol | 802 | ere(B) | MLSB |
| 912 | fexA | Amphenicol | 803 | erm(A) | MLSB |
| 913 | floR | Amphenicol | 804 | erm(B) | MLSB |
| 1538 | optrA | Amphenicol | 805 | erm(D) | MLSB |
| 24 | cfiA | Beta_Lactam | 806 | erm(E) | MLSB |
| 28 | blaACC-1 | Beta_Lactam | 807 | erm(G) | MLSB |
| 34 | blaMOX/blaCMY | Beta_Lactam | 808 | erm(O) | MLSB |
| 35 | blaOCH | Beta_Lactam | 809 | erm(Q) | MLSB |
| 36 | blaPAO/PDC | Beta_Lactam | 810 | erm(S) | MLSB |
| 38 | blaVEB | Beta_Lactam | 811 | lnuB | MLSB |
| 39 | bla1 | Beta_Lactam | 812 | mphA | MLSB |
| 40 | blaOKP | Beta_Lactam | 813 | vat(A) | MLSB |
| 41 | blaROB | Beta_Lactam | 814 | erm(34) | MLSB |
| 42 | blaOXY-2 | Beta_Lactam | 815 | erm(35) | MLSB |
| 43 | blaPSE | Beta_Lactam | 816 | erm(42) | MLSB |
| 46 | cphA | Beta_Lactam | 817 | erm(F) | MLSB |
| 48 | bla-L1 | Beta_Lactam | 818 | lnu(F) | MLSB |
| 106 | cfxA | Beta_Lactam | 819 | lsa(C) | MLSB |
| 107 | cepA | Beta_Lactam | 820 | mef(B) | MLSB |
| 108 | blaCMY | Beta_Lactam | 823 | msr(D) | MLSB |
| 112 | ampC/blaDHA | Beta_Lactam | 824 | msr(E) | MLSB |
| 120 | blaGES | Beta_Lactam | 826 | vgaA | MLSB |
| 121 | blaSFO | Beta_Lactam | 827 | vga(A)LC | MLSB |
| 122 | blaTLA | Beta_Lactam | 828 | vatB | MLSB |
| 123 | blaZ | Beta_Lactam | 1511 | mefA | MLSB |
| 147 | blaVIM | Beta_Lactam | 1519 | lnuC | MLSB |
| 153 | Pbp5 | Beta_Lactam | 79 | nisB | Other |
| 154 | pbp | Beta_Lactam | 239 | pmrA | Other |
| 155 | mecA | Beta_Lactam | 700 | fosb | Other |
| 162 | blaCTX-M | Beta_Lactam | 702 | fosX | Other |
| 236 | penA | Beta_Lactam | 703 | Arr2 | Other |
| 324 | imp-marko | Beta_Lactam | 704 | mcr-1 | Other |
| 362 | NDM new | Beta_Lactam | 1500 | bacA_F | Other |
| 1104 | beta_ccra | Beta_Lactam | 1520 | fabK | Other |
| 1105 | cefa_ampc | Beta_Lactam | 1542 | ARR-3 | Other |
| 1107 | bl1acc | Beta_Lactam | 1543 | mcr-2 | Other |
| 1108 | blaCTX-M-1,3,15 | Beta_Lactam | 133 | sul2 | Sulfonamide |
| 1110 | blaSHV-11 | Beta_Lactam | 177 | strB | Sulfonamide |
| 1113 | bl3_cpha | Beta_Lactam | 208 | folA | Sulfonamide |
| 1114 | blaB-11,13,14 | Beta_Lactam | 280 | sulA/folP | Sulfonamide |
| 1115 | blaIND | Beta_Lactam | 363 | sul1 NEW | Sulfonamide |
| 1116 | blaLEN | Beta_Lactam | 705 | sulIII-marko | Sulfonamide |
| 1118 | blaOXY-1 | Beta_Lactam | 1530 | Enterococci | Taxanomic |
| 1119 | bla-SME | Beta_Lactam | 1531 | Pseudomonas aeruginosa | Taxanomic |
| 1120 | blaCARB | Beta_Lactam | 1532 | Staphylococci | Taxanomic |
| 1121 | blaGOB | Beta_Lactam | 1534 | K. pneumoniae | Taxanomic |
| 1122 | blaHERA | Beta_Lactam | 1535 | A. baumannii | Taxanomic |
| 1123 | blaMIR | Beta_Lactam | 1537 | Campy | Taxanomic |
| 1125 | blaFOXnew | Beta_Lactam | 1554 | Bacteroidetes | Taxanomic |
| 1127 | nonmobile_blaADC | Beta_Lactam | 1555 | Firmicutes | Taxanomic |
| 1128 | nonmobile blaBEL | Beta_Lactam | 294 | tetbP | Tetracycline |
| 1129 | blaIMI | Beta_Lactam | 500 | tet40 | Tetracycline |
| 1505 | ampC | Beta_Lactam | 501 | tetD | Tetracycline |
| 1506 | blaOXA10 | Beta_Lactam | 502 | tetPB | Tetracycline |
| 1508 | blaIMIR | Beta_Lactam | 505 | tet39 | Tetracycline |
| 1512 | blaTEM | Beta_Lactam | 506 | tetG_F | Tetracycline |
| 1517 | blaPER | Beta_Lactam | 507 | tetR | Tetracycline |
| 1523 | KPC BOB | Beta_Lactam | 22 | tet(36) | Tetracycline |
| 1544 | bla-ACT | Beta_Lactam | 54 | tet(32) | Tetracycline |
| 248 | qnrA | Fluoroquinolone | 69 | tetU | Tetracycline |
| 328 | qnrB-bob_redesign | Fluoroquinolone | 180 | tetA | Tetracycline |
| 1200 | norA | Fluoroquinolone | 181 | tetB | Tetracycline |
| 1201 | qepA_1_2 | Fluoroquinolone | 184 | tetK | Tetracycline |
| 1202 | QnrB4 | Fluoroquinolone | 185 | tetQ | Tetracycline |
| 1203 | QnrS1_S3_S5 | Fluoroquinolone | 187 | tetH | Tetracycline |
| 1204 | QnrVC1_VC3_VC6 | Fluoroquinolone | 191 | tetW | Tetracycline |
| 1205 | QnrVC4_VC5_VC7 | Fluoroquinolone | 192 | tetO | Tetracycline |
| 1574 | qnrD | Fluoroquinolone | 195 | tetL | Tetracycline |
| 1576 | qnrS2 | Fluoroquinolone | 196 | tetX | Tetracycline |
| 1577 | oqxA | Fluoroquinolone | 199 | tetC | Tetracycline |
| 1579 | qnrB46,47,48 | Fluoroquinolone | 200 | tetS | Tetracycline |
| 9 | acrB | MDR | 228 | tetV | Tetracycline |
| 11 | acrF | MDR | 291 | tetE | Tetracycline |
| 12 | adeA | MDR | 297 | tetT | Tetracycline |
| 20 | Multidrug resistance protein | MDR | 301 | tet(36) | Tetracycline |
| 57 | cmr | MDR | 1507 | tetPA | Tetracycline |
| 62 | acrA | MDR | 1513 | tetM | Tetracycline |
| 64 | emrD | MDR | 1516 | tetJ | Tetracycline |
| 81 | mdtE/yhiU | MDR | 1518 | tet(38) | Tetracycline |
| 89 | mexA | MDR | 1539 | tet44 | Tetracycline |
| 152 | nimE | MDR | 58 | dfrA1 | Trimethoprim |
| 156 | emrB/qacA | MDR | 59 | dfrA12 | Trimethoprim |
| 231 | mtrE | MDR | 600 | dfra14 | Trimethoprim |
| 234 | oprD | MDR | 601 | dfra17 | Trimethoprim |
| 243 | ttgA | MDR | 602 | dfra7 | Trimethoprim |
| 245 | mepA | MDR | 603 | dfra21 | Trimethoprim |
| 246 | mexE | MDR | 604 | dfra5 | Trimethoprim |
| 253 | mtrD | MDR | 606 | dfrA8 | Trimethoprim |
| 298 | tolC | MDR | 607 | dfrA10 | Trimethoprim |
| 331 | merA-marko | MDR | 608 | dfrA15 | Trimethoprim |
| 355 | marR | MDR | 609 | dfrA18 | Trimethoprim |
| 1300 | mdth | MDR | 610 | dfrA22 | Trimethoprim |
| 1301 | cefa_qacEdelta | MDR | 611 | dfrA25 | Trimethoprim |
| 1302 | mdtg | MDR | 612 | dfrA27 | Trimethoprim |
| 1303 | pcoA | MDR | 613 | dfrAB4 | Trimethoprim |
| 1304 | silE | MDR | 614 | dfrC | Trimethoprim |
| 1305 | arsA | MDR | 615 | dfrG | Trimethoprim |
| 1306 | qacA/B | MDR | 616 | dfrK | Trimethoprim |
| 1308 | qacF/H | MDR | 617 | dfrBmulti | Trimethoprim |
| 1504 | bexA/norM | MDR | 223 | vanXB | Vancomycin |
| 1509 | mdtA | MDR | 306 | vanRB | Vancomycin |
| 1529 | copA | MDR | 307 | vanRC | Vancomycin |
| 1536 | czcA | MDR | 308 | vanRC4 | Vancomycin |
| 1549 | sugE | MDR | 309 | vanRD | Vancomycin |
| 1571 | tcrB | MDR | 311 | vanSC | Vancomycin |
| 1572 | terW | MDR | 313 | vanSE | Vancomycin |
| 1573 | pbrT | MDR | 314 | vanTE | Vancomycin |
| 1575 | cadC | MDR | 315 | vanTC | Vancomycin |
| 1578 | adeI | MDR | 316 | vanTG | Vancomycin |
| 211 | VanB | Vancomycin | 317 | vanYB | Vancomycin |
| 213 | vanD | Vancomycin | 318 | vanYD | Vancomycin |
| 214 | vanHD | Vancomycin | 1002 | vanG | Vancomycin |
| 215 | vanHB | Vancomycin | 1003 | vanC2/vanC3 | Vancomycin |
| 216 | vanRA | Vancomycin | 1514 | vanA | Vancomycin |
| 218 | vanSA | Vancomycin | 1515 | vanXA | Vancomycin |
| 220 | vanWB | Vancomycin | 1521 | vanSB | Vancomycin |
